# Correction: Investigating the effectiveness of structured mindfulness sessions in mitigating burnout among final-year dental students: A mixed-methods analysis

**DOI:** 10.1371/journal.pone.0344206

**Published:** 2026-03-02

**Authors:** Aliya Islam, Javeria Rehman, Sumbul Mujeeb, Rubab Jawed, Unaiza Hashmi, Tazeen Saeed Ali

The fourth author’s name is spelled incorrectly. The correct name is: Rubab Jawed. The correct citation is: Islam A, Rehman J, Mujeeb S, Jawed R, Hashmi U, Ali TS (2026) Investigating the effectiveness of structured mindfulness sessions in mitigating burnout among final-year dental students: A mixed-methods analysis. PLoS One 21(2): e0342017. https://doi.org/10.1371/journal.pone.0342017.
