# Supplementary material for: Investigating the effectiveness of structured mindfulness sessions in mitigating burnout among final-year dental students: A mixed-methods analysis
Source: PLoS One. 2026 Feb 5;21(2):e0342017. doi: 10.1371/journal.pone.0342017 (PMC12875485; doi:10.1371/journal.pone.0342017)
Supplement: Attachment — Submitted filename: Response_to_Reviewers.docx [file pone.0342017.s002.docx]

# Response to Reviewers

We would like to sincerely thank the Academic Editor and both reviewers for their thoughtful and constructive feedback on our manuscript titled “Effectiveness of Mindfulness Practices in Mitigating Burnout Among Final-Year Dental Students.” We have carefully considered each comment and revised the manuscript accordingly. Below, we provide a detailed, point-by-point response to all comments raised by the reviewers and editorial office. Reviewer comments are presented in italics, followed by our responses.

## Editorial Office Comments

***1. Manuscript formatting and file naming***

We have revised the manuscript to comply with PLOS ONE’s formatting requirements, including appropriate file naming conventions. All figures and supporting information files now include captions as per journal guidelines.

***2. Data availability and ethical restrictions***

The Data Availability Statement has been updated accordingly to specify that the datasets contain potentially identifiable information and therefore cannot be made publicly shared due to ethical restrictions by the Baqai Medical University Ethics Review Board.

Qualified researchers may request access to anonymized data by contacting the Ethics Review Board at **registrar.secretariat@baqai.edu.pk**.

(Revised manuscript, ***Page 24, Lines 631–638***)

***3. Non-author contact for data access***

To ensure long-term data accessibility, data requests may be directed to the Baqai Ethical Review Board (IRB) at **registrar.secretariat@baqai.edu.pk,** which serves as the non-author institutional body responsible for reviewing and responding to data requests. All data are securely stored in the institutional repository to ensure persistence.

***4. Ethics statement location***

The ethics statement has been relocated exclusively to the Methods section, in accordance with PLOS ONE requirements.

***5. Figure captions***

Separate captions have been added for each figure, clarifying the content and context of all visual materials.

***6. Supporting Information captions***

Captions for Supporting Information files have been added at the end of the manuscript, and all in-text citations have been updated accordingly.

## Reviewer #1 Comments

1. ***Abstract and introductio****n*

We appreciate positive feedback and have streamlined the abstract to enhance conciseness.

**2- *Consider including a brief explanation about how the dentistry course in Pakistan works - How many years is it? How long are the days? How are they assessed/taught?, This could provide a bit more context for international readers who are unaware***

**Author** **Response:** We thank the reviewer for this suggestion. We have added a short paragraph in the Introduction providing context on the dental education system in Pakistan, including program duration, daily schedules, and assessment methods (Revised manuscript, ***Page 3–4, Lines 81–90*,** highlighted in the revised manuscript).

***Methods clarification***

***1-Explanation of participant selection is a bit unclear, were the 12 participants staff selected to lead the focus groups? Or were these 12 participants the remaining students from the original 69, once you had eliminated those who did not attend sessions, etc***

***Response:*** The qualitative component aimed to gather expert perspectives to inform the development of the Mindfulness-Based Life Skill Building (LSB) curriculum. The 12 participants were **stakeholders**, not students, and were **not part of the burnout assessment cohort** (Revised manuscript, ***Pages 5–6, Lines 144–147***, highlighted in the revised manuscript).

**2- *Why was the CBI-SS tool selected over other tools to measure burnout?***

***Response*:** We have clarified in the Methods section that the CBI-SS was chosen for its reliability, validity, and practicality in assessing multiple dimensions of student burnout (Revised manuscript, ***Page 7, Lines 194–198***, highlighted in the revised manuscript).

**3-*How much time passed between carrying out the first and second mindfulness sessions? Consider exploring in the discussion whether this may have had an effect on burnout levels?***

**Response:** Both sessions were held on the same day, with a 45-minute break between sessions (Revised manuscript, ***Page 8, Lines 215–219***). We have also acknowledged in the Limitations section that conducting only two sessions on the same day may have influenced the observed burnout outcomes (Revised manuscript, **Page 23, Lines 592–595,** highlighted in the revised manuscript).

***Results section***

**1-*What was the overall participation rate? It is mentioned that some students were excluded (eg, because they did not attend both mindfulness sessions), overall, what % of final year dental students participated in the full study? - if low, mention the limitations of this in the discussion.***

**Response:** All final-year dental students attended both mindfulness LSB sessions and completed the pre- and post-intervention assessments, resulting in a 100% participation rate. Although exclusion criteria were defined (students unwilling to participate or absent from more than one session), no participants met these criteria; therefore, no students were excluded from the analysis ***(Lines 156–160, Page 6).***

Regarding the suggestion to discuss limitations related to participation rate, this has been adequately addressed in our existing discussion of scope and generalizability in the limitations section ***(Lines 586–588, Page 22-23).***

**2*-Some of the themes seemed wordy and overly descriptive. I didn’t feel that describing dental students as “weary sailors” helped to contribute towards the value of this research***

**Response**: We have revised the phrasing in the results section ***(Lines 317–320, Page 11)*** to adopt a more concise and objective tone, replacing figurative language such as “weary sailors” with terminology more aligned to academic reporting.

***3- Similar to the above point - line 538 in the discussion - “provided a rich texture” feels overly descriptive and not in keeping with the nature of the paper***

**Response:** The phrase “provided a rich texture” has been rephrased in the discussion section ***(Lines 540–541, Page 21)***

***Discussion section***

*1-****Are there any changes that could be made to the focus group to have a more significant effect on colleague and teacher-related burnout? Or can this only be achieved by changes made to the faculty?***

**Response***:* The focus group in this study was conducted as a preliminary qualitative component to inform the feasibility, content, and structure of the Mindfulness-Based Life Skill Building (LSB) sessions, rather than as part of the intervention itself.

The mindfulness intervention primarily targeted individual coping mechanisms and emotional regulation, which significantly reduced personal and study-related burnout. However, colleague- and teacher-related burnout are relational and context-driven constructs, more strongly influenced by institutional culture and faculty dynamics.

Therefore, meaningful change in these domains would likely require faculty- or system-level interventions rather than modifications to the focus group process. This distinction has been clarified in the revised manuscript

**2-*Little mention of the limitations of this study, this is a key area to consider, especially as burnout and stress can be so subjective and vary massively between individuals (eg: this was only carried out on students in a specific year group at a specific dental school, did the time of year influence the burnout levels of students, ie were the higher burnout levels recorded close to exam periods?)***

**Response:** A detailed Limitations section has been ***added (Pages 22–23, Lines 583–602)*** that discusses factors such as the single-institution sample, restriction to final-year students, the short-term data-collection period, and the timing of data collection that may have influenced burnout levels.

***3-Burnout is quite a sensitive topic, consider whether students may have been under- or over- reporting their experiences and how this may pose a limitation?***

**Response**: Burnout is a sensitive and subjective topic, and students’ self-reported experiences may have been influenced by under- or over-reporting, introducing potential bias. Moreover, burnout levels may vary depending on the time of year, such as around exam periods, which was not accounted for in this study. These factors suggest caution when generalizing the results and highlight the need for future studies to address these variables.
These limitations have been discussed in detail in the revised ***manuscript (Pages 23, Lines 597–600)*** and highlighted.

## Reviewer #2 Comments

**1-*Burnout (BO) needs to be framed as a syndrome in the manuscript. According to the World Health Organization and based on the seminal work of Dr. C. Maslach and Dr. S.E. Jackson, burnout syndrome results from prolonged exposure to chronic stress. Providing detailed background information on burnout syndrome is important as it relates to the manuscript’s title and key words the authors chose.***

**Response**: The manuscript has been revised to consistently frame burnout as **burnout syndrome (BO)**, with a detailed background provided based on the seminal work of Maslach and Jackson and the World Health Organization classification. These changes have been incorporated in the Introduction section (***Pages 3, Lines 60–68***, highlighted in the revised manuscript) to provide clarity and context for international readers and to align with the manuscript title and keywords.

**2*-Based on how the literature defines “efficacy” this study design did not appear to meet standards to measure the efficacy of the mindfulness-based therapies that were examined in this study. For example, the study is lacking randomized control trials and a robust standardization of the intervention protocol to help control for variability in implementation to prevent bias from influencing results. Perhaps the term “effectiveness” might be more appropriate for this study rather than the term efficacy.***

**Response:** We agree with the reviewer that “effectiveness” is a more appropriate term for our quasi-experimental study design, which did not include randomization or full standardization of the intervention. Accordingly, the term “effectiveness” has been updated throughout the manuscript, including the title and abstract, to accurately reflect the scope of our study.

***3-The participants of this study were all from one dental school-- Baqai Dental College in Karachi, Pakistan. A multiple center study to include more schools and final-year dental students would provide a more robust data set because a larger sample size will allow for more statistical power in the data analysis methods. A sample size of 69 final-year dental students from one dental school cohort are too few to publish “efficacy” data on the mindfulness intervention examined in this study. Perhaps it might be more appropriate to frame the study as a pilot study instead.***

**Response***:* We appreciate the reviewer’s insightful comment. While this study was conducted at a single institution with one cohort, the primary aim was to examine the **effectiveness** of mindfulness-based life skill-building sessions using the CBI-SS, applied for the ***first time in Pakistan***. We therefore consider this a full investigation rather than a pilot study. Nonetheless, we acknowledge the single-institution design and sample size as limitations, and this has been clarified in the revised manuscript.

***4-There should be discussion and quantitative statistical analysis to examine if (or to what extent) the demographic data collected -- such as gender -- may have influenced the study’s pre-survey and post-survey burnout findings. For example, there is evidence in the literature that female students tend to experience more stress and burnout when compared to their male counterparts.***

**Response:** While demographic data, including age, gender, institution, and academic year, were collected, the current study focused on assessing burnout among the overall population of final-year dental students rather than performing subgroup analyses, which were beyond the scope of this study. We acknowledge that examining the influence of demographics such as gender on burnout is an important area for future research, which could provide a more nuanced understanding of factors contributing to burnout in dental education.

***4-Description of the Copenhagen Burnout Inventory (CBI-SS)***

**Response**: We have expanded the Methods section to provide details on the CBI-SS subscales, number of items, scoring scale, and reverse scoring, ensuring clarity for readers unfamiliar with the instrument (***Pages 7, Lines 200–207,*** highlighted).

***Overall comment***

- We appreciate the reviewer’s recognition of the study’s potential and have refined the manuscript to improve clarity, methodological justification, and alignment with journal expectations.
- We once again thank the Academic Editor and both reviewers for their valuable time and insightful comments. We believe the manuscript has significantly improved following these revisions and hope it will now meet the journal’s publication standards.
