# Supplementary material for: Investigating the effectiveness of structured mindfulness sessions in mitigating burnout among final-year dental students: A mixed-methods analysis
Source: PLoS One. 2026 Feb 5;21(2):e0342017. doi: 10.1371/journal.pone.0342017 (PMC12875485; doi:10.1371/journal.pone.0342017)
Supplement: Attachment — Submitted filename: Response to Reviewers and Academic Editor.docx [file pone.0342017.s003.docx]

**Response to Reviewers and Academic Editor**
Manuscript ID: PONE-D-25-38768R1
Title: “Investigating the effectiveness of structured mindfulness sessions in mitigating burnout among final-year dental students: A mixed-methods analysis.

We sincerely thank the Academic Editor and reviewers for their constructive comments and suggestions. We have carefully addressed each point and revised the manuscript accordingly. Below is a point-by-point response:

**Editor Comments and Responses**

**1. “Throughout the manuscript p=0.000 should be replaced with p<0.001; additionally, please provide exact p values for those values >0.001.”**
**Response:**

- All instances of “p=0.000” have been replaced with “p<0.001” throughout the manuscript.
- Exact p-values have been reported to three decimal places for all values greater than 0.001.
- These changes have been applied in the text, tables, and figures.

**2. “The number of decimal places for numerical data adjusted to match an appropriate level of precision, for example SD would normally only be reported to 2 decimal places. For most data a maximum of 2 decimals would be appropriate (except p values which have 3).”**
**Response:**

- All numerical values have been revised to use a maximum of 2 decimal places for means, standard deviations, and standard errors.
- P-values are reported with 3 decimal places as recommended.
- This adjustment has been applied consistently across all tables and the main text.

**3. “Please remove colour from Tables and check labels are all correct and in full (i.e. not abbreviated form that may have been used during analysis).”**
**Response:**

- All tables have been reformatted to remove colour.
- Table labels and headings have been checked and expanded to use full, descriptive names (e.g., “Personal Burnout” instead of “PB”; “Teacher-Related Burnout” instead of “TRB”).
- All tables now comply with PLOS ONE formatting guidelines.

**4. “Please do not refer to differences where p>0.05 as tends, showing improvement in positive direction or indicative of differences. If the p-value does not meet your pre-determined threshold, then it is appropriate to make statements that imply differences.”**
**Response:**

- Text referring to non-significant differences (p>0.05) has been revised.
- Phrases such as “tends to improve” or “indicative of differences” have been removed.
- Statements now reflect that changes were **not statistically significant** without implying an effect.

**5. “Suggest moderating the language for the manuscript to avoid use of phrases such as ‘A bold and imperative objective …’ or ‘… there were alarmingly high levels of exhaustion …’.”**
**Response:**

- Language throughout the manuscript, including the Discussion and Results sections, has been reviewed and moderated to maintain a neutral, scientific tone.
- Emotive or emphatic phrases (e.g., “A bold and imperative objective” or “alarmingly high levels of exhaustion”) have been replaced with descriptive, objective alternatives that accurately convey the findings without overstatement.

**6. References and citations**
**Response:**

- The reference list has been reviewed for completeness and accuracy.
- No retracted articles are cited; all references are current and relevant.
- Changes to the reference list have been reflected in the manuscript as needed.

**7. Figures and technical requirements**
**Response:**

- All figures were reviewed for compliance with PLOS ONE guidelines, including resolution, format, labeling, and clarity.
- Two figures required minor adjustments, which have been made and the updated versions have been uploaded.
- The remaining figures were checked and confirmed to meet the journal’s technical requirements.

**Summary:**
All comments from the Academic Editor have been addressed, and the manuscript has been revised accordingly. We thank the Editor and reviewers for their guidance, which has helped improve the clarity, precision, and overall quality of our manuscript.

We hope that the revised manuscript meets the requirements for publication in PLOS ONE.
